# Idiopathic sudden sensorineural hearing loss in dialysis patients

**DOI:** 10.1080/0886022X.2018.1450760

**Published:** 2018-03-22

**Authors:** Sun-Myoung Kang, Hyun Woo Lim, Hoon Yu

**Affiliations:** a Division of Nephrology, Department of Internal Medicine, Asan Medical Center, University of Ulsan College of Medicine, Seoul, Korea;; b Department of Otolaryngology-Head and Neck Surgery, Gangneung Asan Hospital, University of Ulsan College of Medicine, Gangneung, Korea;; c Division of Nephrology, Department of Internal Medicine, Gangneung Asan Hospital, University of Ulsan College of Medicine, Gangneung, Korea

**Keywords:** Hearing loss, dialysis, chronic kidney disease, renal failure

## Abstract

Although sudden sensorineural hearing loss (SSNHL) affects chronic kidney disease (CKD) patients more frequently than non-CKD patients, few reports have described SSNHL in dialysis patients. We aimed to review the characteristics of SSNHL in chronic dialysis patients and evaluate treatment responses to steroid therapy. We retrospectively reviewed the records of dialysis patients diagnosed with idiopathic SSNHL at Asan Medical Center between January 2000 and December 2014. Pure tone and speech audiometry analyzes were performed before and 2 weeks and 2 months after treatment onset to evaluate outcomes. Twenty-two patients (11 men, 11 women; mean age: 49.9 ± 11.7 years) were included; 16 (72%) and 6 (28%) had undergone hemodialysis and peritoneal dialysis, respectively, for a median of 49.2 ± 41.4 (1–144) months. End-stage renal disease was most frequently caused by diabetic nephropathy (11 cases), chronic glomerulonephritis (1 case) and unknown factors (7 cases). Common accompanying symptoms included tinnitus (68.2%), ear fullness (45.5%) and vertigo (27.3%). The mean pure tone audiometry threshold at the initial presentation was 82.6 ± 22.4 dB. At 2 months post-steroid treatment, 4 (18.2%), 4 (18.2%) and 6 (27.3%) patients exhibited a complete, partial, or slight recovery, respectively; 8 patients (36.3%) showed no improvement. Although we could not identify the specific cause of SSNHL in this population, our relatively large case series elucidates the precise clinical features of SSNHL in this population and demonstrates the outcomes of steroid treatment.

## Introduction

Sudden sensorineural hearing loss (SSNHL) is defined as a hearing loss of ≥30 dB at three sequential frequencies occurring over a 72-h period. Currently, although no strong evidence supports the efficacy of any treatment option for SSNHL, glucocorticoids are generally considered the first-line therapy. Glucocorticoids can be administered systemically or via intratympanic installation; the latter is often used when hearing does not improve after systemic therapy and can also be used as an initial therapy for patients in whom high-dose systemic glucocorticoids administration should be avoided (e.g., those with diabetes mellitus).

Idiopathic SSNHL (ISSNHL) frequently occurs in patients with chronic kidney disease (CKD), and its incidence is higher among this population relative to non-CKD patients [1]. Renal failure might contribute to sensorineural hearing loss (SNHL) via factors such as the osmotic alterations caused by hemodialysis, similarities in antigenicity between the labyrinths and the kidney, uremic neuropathy and ototoxins [2]. However, the etiology of SSNHL in this population remains unclear, as only a few studies and case reports regarding SSNHL in dialysis patients have been published [2,3]. Therefore, we reviewed the development of SSNHL in chronic dialysis patients to study the characteristics of this condition and evaluate the responses to steroid treatment in this population.

## Patients and methods

### Patients

This study was approved by the Institutional Review Board of Asan Medical Center (S2015–1928-0001). We retrospectively reviewed the medical records of patients diagnosed with idiopathic SSNHL in dialysis patients at Asan Medical Center between January 2000 and December 2014. Asan Medical Center is a superior general hospital. Approximately 15,000 and 2,000 patients visited this hospital to receive hemodialysis and peritoneal dialysis, respectively, from 2000 to 2014.

All diagnoses of SSNHL were made via pure tone audiometry according to the American Academy of Otolaryngology-Head and Neck Surgery (AAO-HNS) practice guideline criteria [4], which mainly comprised the following: (1) acute onset of SNHL over a 72-h period, and (2) audiometric confirmation of a 30-dB hearing loss at three consecutive frequencies. Patients were excluded if they had other causes of SNHL (e.g., Meniere’s disease), a history of chronic otitis media or otologic surgery, symptoms or signs of central disorders or abnormal MR imaging findings, or poorly reliable audiometric test data. We reviewed the demographic, audiologic, treatment and dialysis data of all included patients.

### Treatment of SSNHL

All enrolled patients had received systemic steroid therapy and/or intratympanic steroid injection. Systemic steroid therapy was considered the first-line treatment for SSNHL and comprised a single morning dose of oral methylprednisolone each day for 2 weeks (48 mg for 9 days, followed by five consecutive days of tapering). Intratympanic steroid injection was administered as an initial therapy for patients who refused systemic steroid therapy or had severe diabetes mellitus or as a salvage therapy for patients who did not exhibit complete or partial recovery after initial 2 weeks of systemic steroid treatment. Intratympanic steroid injections of 5 mg dexamethasone were administered twice weekly for two consecutive weeks. Additional treatments, including antiviral agents, prostaglandin therapy, vitamins and hyperbaric oxygen therapy were rarely used as second line treatments.

### Outcome evaluations and data analysis

Pure tone audiometry and speech audiometry tests were performed before treatment and 2 weeks and 2 months after treatment onset to evaluate the outcomes. The pure tone average was calculated by measuring 4 frequencies (0.5, 1, 2 and 3 kHz). The speech audiometry results were analyzed to compare the pretreatment and post-treatment speech discrimination scores (SDSs). Treatment responses were defined as complete, partial, slight, or no improvement according to Siegel’s criteria (Table 1) [5]. All statistical analyzes were performed using IBM SPSS Statistics for Windows, version 23.0 (IBM Corp., Armonk, NY). *p* values <.05 were considered to indicate statistically significant differences.

**Table 1.** Improvement criteria used in this study (Siegel’s criteria).

	Type	Hearing recovery
I.	Complete recovery	Patients whose final hearing level was better than 25 dB regardless of the size of the gain
II.	Partial recovery	Patients who showed more than 15 dB of gain and whose final hearing level was between 25 and 45 dB
III.	Slight improvement	Patients who showed more than 15 dB of gain and whose final hearing level was poorer than 45 dB
IV.	No improvement	Patients who showed less than 15 dB of gain

## Results

Twenty-two patients (11 men, 11 women, mean age: 49.9 ± 11.7 years) were included in this study, and their clinical characteristics are described in Table 2. The right and left sides were involved in 11 patients each. Sixteen patients (72.7%) had undergone hemodialysis and 6 (27.3%) had undergone peritoneal dialysis for a median of 35 (1–144) months. The diseases predisposing patients to end-stage renal disease were diabetic nephropathy in 11 cases (50%), chronic vasculitis in 1 case (4.5%), chronic glomerulonephritis in 1 case (4.5%), polycystic kidney disease in 1 case (4.5%), systemic lupus erythematosus in 1 case (4.5%) and unknown in 7 cases (32.0%).

**Table 2.** Clinical characteristics.

	Number of patients (*n* = 22)
Age (mean ± SD, years)	49.9 ± 11.7
Gender (Male:Female)	11:11
Affected ear (Left:Right)	11:11
Time from onset of symptoms of SSNHL to treatment	
Mean ± SD	3.9 ± 3.3
Range	0–12
Underlying disease	
Diabetes mellitus (%)	13 (59.1)
Hypertension (%)	16 (72.7)
Associated symptom^a^	
Tinnitus (%)	15 (68.2)
Ear fullness (%)	10 (45.5)
Vertigo (%)	6 (27.3)
Dialysis type	
Hemodialysis (%)	16 (72.7)
Peritoneal dialysis (%)	6 (27.3)
Cause of ESRD (%)	
Diabetic nephropathy (%)	11 (50.0)
Chronic glomerulonephritis (%)	1 (4.5)
Polycystic kidney disease (%)	1 (4.5)
Vasculitis (%)	1 (4.5)
Lupus nephritis (%)	1 (4.5)
Unknown (%)	7 (32.0)

a These conditions are not mutually exclusive.

The following symptoms were associated with SSNHL: tinnitus in 15 cases (68.2%), ear fullness in 10 cases (45.5%) and vertigo in 6 cases (27.3%). Loop diuretics and other nephrotoxic medications were not administered to the enrolled patients before the onset of SSNHL.

The mean pure tone audiometry threshold of the affected side at the initial presentation was 82.6 ± 22.4 dB (Table 3). The mean pure tone audiometry threshold of the unaffected side at the initial presentation was 20.88 ± 12.3 dB.

**Table 3.** Audiometric results according to times (*n* = 22).

	Initial	Post-treatment 2 weeks	Post-treatment 2 months
Affected side PTA (dB)			
Mean ± SD	82.6 ± 22.4	62.3 ± 31.0	57.6 ± 29.2
Range	36.3–118.8	6.3–120.0	6.3–111.3
Affected side SDS (%)			
Mean ± SD	27.5 ± 35.4	43.8 ± 42.9	51.3 ± 39.4
Range	0–92	0–100	0–100

**Figure 1.**
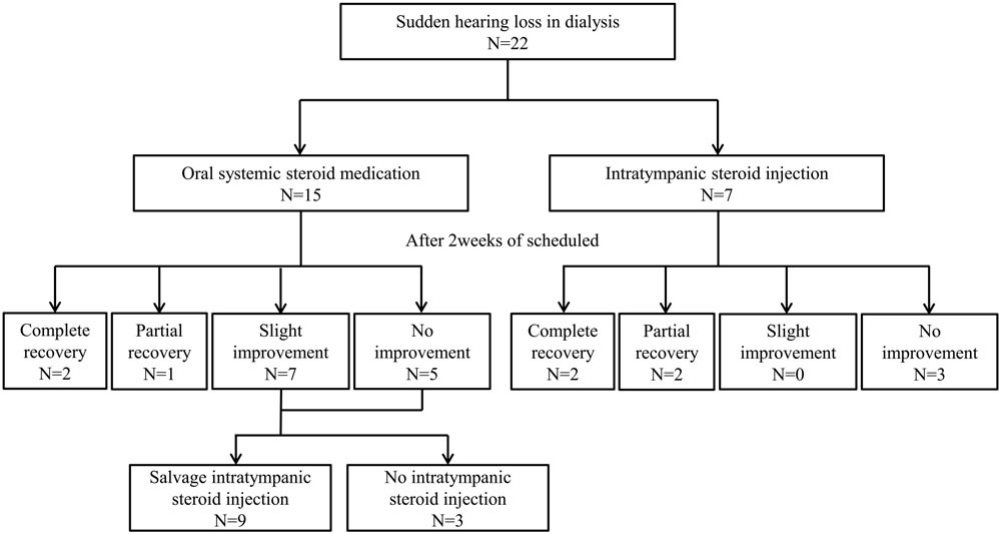
Treatment flow of enrolled patients.

Initially, 15 patients were treated with oral systemic steroid medication and 7 were treated with intratympanic steroid injection. Among those receiving systemic steroid therapy, 9 patients received additional intratympanic steroid injections as salvage therapy after completing a 2-week course of systemic steroid medication (Figure 1). After 2 weeks of steroid treatment, 4 (18.2%) and 3 of 22 (13.6%) patients achieved a complete or partial hearing recovery, respectively, and 7 (31.8%) achieved slight recovery. In contrast, 8 of 22 patients (36.4%) showed no improvement. After 2 months of steroid treatment, 4 (18.2%) and 4 of 22 (18.2%) patients had achieved complete or partial hearing recovery, respectively and 6 (27.2%) had achieved a slight recovery. Furthermore, 8 of 22 patients (36.4%) showed no improvement (Table 4).

**Table 4.** Hearing improvement according to Siegel’s criteria at 2 weeks and 2 months follow-up.

	2 weeks after treatment (*n* = 22)	2 months after treatment (*n* = 22)
Complete recovery	4 (18.2%)	4 (18.2%)
Partial recovery	3 (13.6%)	4 (18.2%)
Slight recovery	7 (31.8%)	6 (27.2%)
No improvement	8 (36.4%)	8 (36.4%)
Complete and partial recovery	7 (31.8%)	8 (36.4%)
Complete, partial and slight improvement	14 (63.6%)	14 (63.6%)

## Discussion

This study investigated cases of SSNHL in 22 patients undergoing dialysis for CKD. After 2 months of steroid treatment, 8 of 22 (36.4%) patients achieved either a complete or partial recovery. However, this treatment outcome among dialysis patients with CKD was worse than the outcomes observed in the general population [6,7]. Although the exact incidence of SSNHL is uncertain, the estimated incidence is roughly 2–20 cases per 100,000 person-years [4,8,9]. Although the exact incidence of SSNHL has also not been determined in CKD patients, Charlene et al. reported a 1.57-fold higher incidence among CKD patients relative to non-CKD controls [1].

Physiologically, the kidney and cochlea play similarly important roles in fluid and electrolyte regulation and may share a common antigenicity. These factors may explain the similar effects of medications and immunological factors on both organs and strongly supports a relationship between hearing disorders and CKD [10]. Several etiological factors, including uremia, ototoxic medication, electrolyte disturbances and hemodialysis treatment, have been linked to hearing disorders in patients with renal failure. Alder et al. reported an inverse correlation between serum creatinine levels and sodium–potassium activated ATPase levels and suggested that the inhibition of this enzyme system might cause inner ear dysfunction in terminal patients with uremia [11].

Several studies have suggested that hemodialysis is a risk factor for the development of SNHL. Kligerman et al. [12] reported high-frequency hearing impairments in patients treated with hemodialysis and identified that a long duration of hemodialysis is a potential contributor to hearing loss. Johnson and Mathog [13] observed hearing fluctuations during single dialysis, as well as high-frequency hearing deficits early in the course of hemodialysis. Furthermore, Rizvi and Holmes reported one case of a patient undergoing peritoneal and hemodialysis who experienced hearing loss, and proposed osmotic disequilibrium associated with hemodialysis as the etiological factor [14].

In contrast, other reports have suggested that SNHL is not related to hemodialysis. Ozturan and Lam studied 15 subjects and 10 controls to evaluate the effects of hemodialysis treatment on hearing loss and found no difference in the pure-tone audiometry test results before and after treatment. The authors concluded that hemodialysis is safe with respect to hearing function [15]. Furthermore, Kusakari et al. analyzed 37 hemodialysis patients to determine the potential effects of hemodialysis and other factors on SNHL. During hearing tests conducted soon after hemodialysis initiation and every 3–12 months thereafter, only four cases exhibited significant hearing loss after hemodialysis. The authors therefore concluded that hemodialysis did not affect hearing functions [16]. In our study, we could not identify a direct relationship between dialysis and SSNHL because no patients developed SSNHL during or immediately after dialysis.

To date, glucocorticoids have been the most widely accepted treatment option for idiopathic SSNHL. Similarly, corticosteroids were principally prescribed for the patients in our study, and no severe adverse effects were reported among those treated with either systemic or intratympanic steroid treatment. According to the published literature, the partial and complete post-treatment recovery rates from SSNHL range between 60 and 73% in general populations [17–20]. Regarding dialysis patients, Makita et al. [21] reported 30 hemodialysis patients from case studies and found that 18 (60%) had recovered completely or partially, whereas 12 (40%) had not recovered. In our study, the partial or complete recovery rate of SSNHL was 36.4% at 2 months after treatment. Compared with the findings of previous studies, our results suggest a somewhat worse prognosis. Factors such as underlying disease, treatment modality and dialysis itself might have contributed to the poor outcome, although further research, including a comparison with a general population, is needed to clarify these issues.

## Conclusion

SSNHL is a difficult complication that affects the quality of life of patients with chronic renal failure. Uremia, ototoxins, anemia, osmotic alteration caused by hemodialysis and similar antigenicities of the cochlear labyrinths and the kidney have been suggested as possible etiologic factors for this condition. Although we could not identify the specific cause of SSNHL in patients undergoing dialysis, our report included relatively many more cases than did previous reports. Furthermore, we elucidated the precise clinical features of SSNHL in dialysis patients. A prospective study will be required to clarify the effects of treatment and the relationship between sudden hearing loss and chronic renal failure.
